# A short history of *Auto-Rickshaw*

**DOI:** 10.1107/S2059798326008831

**Published:** 2026-09-22

**Authors:** Santosh Panjikar, Victor Lamzin, Paul A. Tucker, Manfred S. Weiss

**Affiliations:** a ANSTO, Australian Synchrotron, 800 Blackburn Road, Clayton, Victoria3168, Australia; b Department of Biochemistry and Molecular Biology, Monash University, Victoria3800, Australia; c European Molecular Biology Laboratory, Hamburg Unit, Hamburg, Germany; d European Molecular Biology Laboratory, Heidelberg and Hamburg Units, Germany; e Macromolecular Crystallography, Helmholtz-Zentrum Berlin, Albert-Einstein-Strasse 15, 12489Berlin, Germany; University of Manchester, United Kingdom

**Keywords:** *Auto-Rickshaw*, experimental phasing, molecular replacement, MRSAD, *SHELX*, macromolecular crystallography, structural biology, *SHELXC*, *SHELXD*, *SHELXE*, synchrotron beamlines, automation

## Abstract

*Auto-Rickshaw* was developed in the early 2000s at EMBL Hamburg as an automated macromolecular structure-determination platform for crystallo­graphy and structural biology. Its initial motivation was to provide beamline users with rapid feedback on whether diffraction data were sufficient for structure solution. The platform was not merely a wrapper around existing crystallographic programs; it encoded the expert decisions needed to select phasing, density-modification, model-building and refinement protocols according to the information available from the experiment. Since its first publication in 2005, *Auto-Rickshaw* has supported a broad range of experimental and model-based strategies, including SAD, MAD, SIR, SIRAS, RIP, molecular replacement and hybrid approaches such as MRSAD. A central feature was the integration of the *SHELX* programs, particularly *SHELXC*, *SHELXD* and *SHELXE*, whose speed, robustness and command-line design made them especially suitable for automated pipelines. This review gives a brief historical account of *Auto-Rickshaw*, the beamline context in which it emerged, the design principles that shaped it and the role of *SHELX* programs in its phasing architecture. It also considers the broader legacy of *Auto-Rickshaw* as a bridge between crystallographic expertise and automated, reproducible structure solution. Finally, the review places *Auto-Rickshaw* in the current landscape of synchrotron technologies, high-throughput structural biology, containerized deployment, predicted structural models and AI-assisted decision making, where reliable automation remains essential.

## Abstract 2

*Auto-Rickshaw* is an example of practical, decision-driven automated macromolecular structure determination from crystallographic diffraction data. This review traces its development at the EMBL Hamburg/DESY beamline environment, where rapid validation of diffraction experiments was essential, through its expansion into a general platform for automated structure solution and its continuing relevance to beamline automation.

## 1. Introduction

For many decades, the crystallographic phase problem constituted the central obstacle to structure determination of biological macromolecules by X-ray crystallography. Diffraction experiments measure reflection intensities, from which structure-factor amplitudes can be derived, but the corresponding phases are not measured directly. Solving a structure therefore requires additional information: anomalous differences, isomorphous differences, radiation-induced changes, prior structural models, noncrystallographic symmetry, density-modification constraints, or some combination of these. The conceptual basis of these approaches is well established, but their practical use has historically required experience, judgement and persistence.

Before the widespread adoption of automated structure-solution pipelines, a crystallographer solving a new macromolecular structure had to make a long sequence of interconnected decisions. Which phasing strategy was appropriate? Was the anomalous signal significant? What resolution cutoff should be used for substructure solution? How many heavy-atom or anomalous-scatterer sites should be expected? Was the substructure hand correct? Was the electron-density map interpretable? Should molecular-replacement phases be combined with anomalous data? When should density modification, model building or refinement be attempted? Each of these choices could determine whether a structure was solved rapidly, solved only after many iterations, or missed entirely.

The development of *Auto-Rickshaw* greatly benefitted from the DESY/EMBL Hamburg macromolecular crystallography (MX) beamline environment, where software automation, user support and experimental decision making were closely interconnected. At the time, EMBL Hamburg operated five MX beamlines at the DORIS/DORIS III storage ring, a second-generation synchrotron source: BW7A, BW7B, X11, X12 and X13. These beamlines provided complementary experimental capabilities: BW7A and later X12 were tuneable beamlines used for anomalous diffraction experiments, whereas BW7B, X11 and X13 primarily supported fixed-wavelength data collection and rapid high-resolution or single-wavelength anomalous diffraction (SAD) experiments. Thus, *Auto-Rickshaw* was developed in an environment that included both wavelength-tuneable experimental phasing beamlines and fixed-wavelength stations (Pohl *et al.*, 2001 ▸, 2004 ▸; Hermes, 2004 ▸).

Moreover, many researchers from the group of George Sheldrick at the University of Göttingen regularly visited EMBL Hamburg for crystallographic experiments and this strongly contributed to the development of methodological innovations.

Data collection at the time was also substantially more beamtime-consuming than in the present era of high-brilliance sources equipped with fast pixel-array detectors. Depending on the crystal, detector, strategy and phasing experiment, a single dataset could require tens of minutes to hours. In that setting, rapid computational validation was not merely a convenience: it could affect the experiment while beamtime was still available, for example by showing that sufficient phasing information had already been collected, or that additional data, a different wavelength or another crystal was required.

*Auto-Rickshaw* was designed as an automated crystal-structure determination platform that could guide diffraction data through alternative phasing and model-building routes. The name itself has a practical origin. In early internal descriptions, the system was referred to more formally as the EMBL Hamburg automated crystal-structure determination platform. During review of the original manuscript, however, the authors were encouraged to give the platform a distinctive name. The term *Auto-Rickshaw* had already been used colloquially within the group because it captured the intended behaviour of the software. In South Asia, an auto-rickshaw, also known in some regions as a tuk-tuk, is a motorized successor to a manually driven rickshaw. By analogy, the program was intended to automate many of the crystallo­graphic decisions that had previously been made manually: choosing routes, responding to obstacles and moving efficiently through a complex structure-solution landscape.

The goal was not to replace crystallographic expertise, but to make expert-like workflows available to non-expert users in a reliable, reproducible and accessible form. This review summarizes the circumstances of its development, the principles behind its architecture, the evolution of its phasing protocols and the role of *SHELX* programs. It follows *Auto-Rickshaw* from a public web service to beamline-integrated, secured and containerized implementations, as summarized in Fig. 1 ▸, and places it alongside contemporary systems such as *SOLVE*/*RESOLVE*, *HKL*-3000, *APRV*, *autoSHARP*, *CRANK* and *ELVES*.

## 2. EMBL Hamburg origins and beamline deployment

*Auto-Rickshaw* was conceived and developed at the EMBL Hamburg Outstation on the DESY campus in Hamburg, a research environment that also served a large international beamline user community. The platform therefore emerged from practical synchrotron needs as much as from software development itself.

At the time, the EMBL Hamburg MX beamlines at DESY formed one of Europe’s major synchrotron MX facilities. BioSync/PDB beamline statistics for deposition years 1995–2009 list more than 2000 publicly available macromolecular PDB depositions under the EMBL/DESY entry, corresponding to approximately one-sixth of all European depositions. EMBL/DESY was the second-largest European synchrotron structure provider, after the third-generation source ESRF (BioSync, 2026 ▸).

During beamtime visits, users often had to decide quickly whether to continue with the current data, collect more data, change strategy or try another crystal. *Auto-Rickshaw* was designed to support those decisions by turning processed diffraction data into rapid phasing attempts, maps, partial models and diagnostic information.

*Auto-Rickshaw* arose from this practical beamline setting. The developers recognized that many parts of expert crystallographic decision making could be encoded as a structured workflow. In its initial form, the pipeline focused primarily on automated experimental phasing from anomalous and derivative data, including SAD, MAD and SIRAS strategies, together with the associated steps of data preparation, substructure determination, phase improvement, density modification, model building and refinement. The system therefore needed to accept user data, analyse the available phasing information, select appropriate software tools, monitor intermediate results and redirect the calculation if necessary (Panjikar *et al.*, 2005 ▸).

The development also belonged to the broader European high-throughput structural biology environment of the early 2000s. Support from the EC-funded BIOXHIT and SPINE projects connected automated structure solution, synchrotron operation and structural genomics (Panjikar *et al.*, 2005 ▸).

The subsequent 2009 introduction of MRSAD, the combination of molecular replacement with SAD phasing, became one of the most important extensions of *Auto-Rickshaw* and is discussed in Section 9 below (Panjikar *et al.*, 2009 ▸).

After the relocation of the principal developer (SP) to the Australian Synchrotron in June 2011, the EMBL Hamburg public web service continued to be maintained and further beamline-focused development proceeded at the Australian Synchrotron, where *Auto-Rickshaw* could be used for rapid data evaluation and user support (Australian Synchrotron, 2011 ▸; Panjikar, 2021 ▸).

As detector readout speed, beamline automation and sample throughput increased, the platform also evolved towards multiple-dataset evaluation. Subsequent developments introduced ‘multiscale’ and ‘multidata’ modes, in which datasets could be selected, clustered, scaled or merged according to unit-cell similarity, space group, isomorphism, radiation damage and anomalous signal before being passed to SAD, SIRAS or MAD phasing workflows (Panjikar *et al.*, 2015 ▸, 2017 ▸). This extended the original single-job validation concept towards beamline-scale data selection and automated evaluation of many related datasets.

The Australian Synchrotron deployment also reflected the wider move towards high-performance and containerized scientific computing, including the 2021 installation on the ASCI cluster using Docker and Kubernetes and the later *ASWEBRICK* (*Australian Synchrotron WEB-based RICKshaw*) development described below. These technologies should be understood as later deployment and orchestration mechanisms rather than as components of the original *Auto-Rickshaw* decision-making methodology.

## 3. Architecture: a web interface coupled to crystallographic decision makers

The original *Auto-Rickshaw* architecture combined a web-based graphical interface with a backend web service that controlled crystallographic programs. Users supplied processed diffraction data, sequence information and experimental details without installing the underlying software locally; the backend connected data preparation, substructure solution, phasing, density modification, model building and refinement.

The system evaluated intermediate outputs and used them to choose subsequent steps. In experimental phasing, the strength of the anomalous or isomorphous signal, substructure quality, hand discrimination, density-modification success and map interpretability could all influence the path forward.

In practical terms, *Auto-Rickshaw* selected among protocols, tested hands or resolution limits, chose downstream programs, stopped weak routes and reported diagnostic evidence. It did not learn during an individual run; its rules evolved through successive releases as new protocols, diagnostics and crystallographic experience were incorporated.

A clear example of this decision logic was the selection of heavy-atom parameter refinement and phase-calculation programs according to quantitative indicators such as the correlation coefficient for the weak signal, solvent content and data resolution. In the original implementation, these criteria were used to choose between alternative downstream programs, including *BP*3 (Pannu & Read, 2004 ▸), *SHARP* (de La Fortelle & Bricogne, 1997 ▸), *MLPHARE* (Winn *et al.*, 2011 ▸) and *SHELXE*, or to halt the calculation when the signal or heavy-atom model was too weak to justify further automated processing (Panjikar *et al.*, 2005 ▸). The precise numerical boundaries between these decision regions were not intended to be immutable; rather, they represented an evolving empirical description of expert crystallographic judgement. This feature is central to the historical importance of *Auto-Rickshaw*: it encoded not only software execution, but also the conditional choices that crystallographers make when assessing whether experimental phasing information is strong enough to proceed.

*Auto-Rickshaw* was made available in two flavours. The beamline, or fast, version was optimized for rapid assessment of diffraction experiments, whereas the advanced version was designed for more complete structure determination and required additional information, including sequence data.

The backend integrated a wide range of crystallographic programs. Data-processing and scaling tools were linked to phasing programs, molecular-replacement programs, density-modification tools, automated model-building programs and refinement packages. The *SHELX* programs occupied a particularly central position in experimental phasing. Molecular replacement was supported through established tools such as *MOLREP* (Vagin & Teplyakov, 1997 ▸) and *Phaser* (McCoy *et al.*, 2007 ▸), while density modification, model building and refinement could involve programs such as *DM* (Winn *et al.*, 2011 ▸), *RESOLVE* (Terwilliger, 2000 ▸), *ARP*/*wARP* (Lamzin & Wilson, 1993 ▸; Langer *et al.*, 2008 ▸), *Buccaneer* (Cowtan, 2006 ▸), *Coot* (Emsley *et al.*, 2010 ▸), *REFMAC* (Murshudov *et al.*, 1997 ▸) and *phenix.refine* (Adams *et al.*, 2010 ▸).

## 4. Phasing strategies supported by *Auto-Rickshaw*

One of the defining features of *Auto-Rickshaw* was the breadth of phasing strategies that it supported. Rather than focusing on a single method, the platform was designed to select among several experimental and model-based routes according to the information available from the diffraction experiment.

Single-wavelength anomalous diffraction (SAD) became one of the most important protocols. In SAD experiments, anomalous differences from selenium, sulfur, halides or other anomalous scatterers provide the information needed to locate a substructure and estimate phases. *Auto-Rickshaw* automated the complete sequence: data preparation and signal analysis, substructure solution, phase improvement, model building and refinement.

Multi-wavelength anomalous diffraction (MAD) was also supported, including experiments with two, three or four wavelengths. MAD experiments were historically central to synchrotron-based macromolecular crystallography. An early landmark was the *de novo* structure determination of a basic blue copper protein from cucumber seedlings using multiple-wavelength anomalous diffraction (Guss *et al.*, 1988 ▸). This and subsequent MAD applications demonstrated the value of tuneable synchrotron beamlines, where data could be collected at peak, inflection and remote wavelengths around an absorption edge. *Auto-Rickshaw* provided a way to manage the additional complexity of multi-wavelength phasing, including wavelength-specific data preparation and substructure determination.

Isomorphous replacement methods, including single isomorphous replacement (SIR) and single isomorphous replacement with anomalous scattering (SIRAS), formed another component of the platform. These methods exploit differences between native and derivative crystals, sometimes combined with anomalous differences. They are sensitive to non-isomorphism and derivative quality, making them well suited to automated diagnostic evaluation.

Molecular replacement (MR) was included because, as the Protein Data Bank expanded, MR became an increasingly dominant route for macromolecular structure solution. This trend was already clear in the late 2000s: the *BALBES* molecular-replacement pipeline was developed to exploit the growing PDB as a search-model repository, and testing of that pipeline indicated that around 75% of all structures could be solved automatically without user intervention (Long *et al.*, 2008 ▸). A later discussion of *MOLREP* noted that about two-thirds of recently deposited X-ray structures in the PDB had been determined by MR (Vagin & Teplyakov, 2010 ▸). *Auto-Rickshaw* therefore included MR programs to obtain initial model phases and then connected these phases to refinement, density modification and automated model building.

Hybrid and specialist routes were also supported. These included molecular replacement combined with SAD phasing (MRSAD), radiation-induced phasing (RIP), molecular-replacement-assisted RIP (MRRIP), sulfur SAD (S-SAD) and later multiple-dataset workflows. These extensions are discussed in more detail in Section 9.

Table 1 ▸ summarizes the phasing methods supported by *Auto-Rickshaw* and the principal *SHELX* programs involved in each route.

## 5. *SHELX* as the main experimental phasing engine

The *SHELX* suite, developed by George M. Sheldrick, became important to *Auto-Rickshaw* because its programs combined algorithmic power with the practical qualities needed for automation: speed, robustness, simple input and output, and command-line operation. The triad *SHELXC*, *SHELXD* and *SHELXE* was especially important for experimental phasing (Sheldrick, 2008 ▸, 2010 ▸; Usón & Sheldrick, 2018 ▸, 2024 ▸).

*SHELXC* provided data preparation and analysis. It estimated anomalous or marker-atom structure-factor amplitudes, generated normalized structure factors and produced input for subsequent *SHELX* programs. Equally importantly, it provided statistical diagnostics that could be used by *Auto-Rickshaw* decision makers. Measures of anomalous correlation and signal strength helped determine whether a dataset was likely to support substructure solution and to what resolution the signal remained useful.

*SHELXD* solved the heavy-atom or anomalous-scatterer substructure using dual-space direct methods (Schneider & Sheldrick, 2002 ▸). In the context of automated pipelines, *SHELXD* was valuable not only because it was powerful, but because it was fast enough to be run repeatedly under different assumptions. *Auto-Rickshaw* could explore alternative resolution cutoffs, expected site numbers or data treatments, increasing the chance of finding a correct substructure without requiring a user to perform trial-and-error searches manually.

*SHELXE* provided phase improvement, density modification and, crucially, automated chain tracing (Sheldrick, 2010 ▸; Usón & Sheldrick, 2018 ▸, 2024 ▸). The ability of *SHELXE* to distinguish the correct hand of a substructure and to produce interpretable density or partial polyalanine models made it an excellent diagnostic and productive step in an automated pipeline. In many cases, the transition from an abstract substructure solution to a map that a crystallographer could recognize as protein-like occurred through *SHELXE*.

More recent developments in *SHELXE* further emphasize why it remains relevant to the *Auto-Rickshaw* philosophy. *SHELXE* can operate with phases derived from experimental data, from partial models or maps, or from combinations of these sources, including workflows such as MRSAD. It can also read external phase values, phase weights or phase-probability distributions encoded as Hendrickson–Lattman coefficients. These capabilities make *SHELXE* well suited to an adaptive pipeline in which phase information may arise from different sources and at different stages of the calculation. The recent extension of tracing into side chains and the explicit treatment of model bias from predicted models also connect *SHELXE* to current developments in *AlphaFold*-assisted molecular replacement and model completion (Usón & Sheldrick, 2024 ▸).

Within *Auto-Rickshaw*, the use of *SHELXE* was therefore not simply automatic in every case. It was selected as part of a broader decision framework in which signal strength, solvent content, resolution and heavy-atom model quality influenced whether *SHELXE*, *MLPHARE*, *BP*3 or *SHARP* was used for subsequent phase calculation or phase improvement (Panjikar *et al.*, 2005 ▸). This historical detail is important because it shows that the central role of *SHELX* in *Auto-Rickshaw* was embedded within a wider adaptive phasing strategy.

The overall experimental phasing workflow is illustrated in Fig. 2 ▸. In this workflow, *SHELX* programs served as dependable engines within a larger decision-making framework: *Auto-Rickshaw* provided the route planning, while *SHELX* provided much of the experimental phasing horsepower.

## 6. *Auto-Rickshaw* in a wider automation landscape

*Auto-Rickshaw* did not emerge in isolation. Its development formed part of a broader movement in macromolecular crystallography towards the automation of data processing, experimental phasing, model building and refinement. Contemporary and near-contemporary systems included *autoSHARP*, *ACrS*, *HKL*-3000, *ELVES*, *APRV*, *CRANK* and *SOLVE*/*RESOLVE*, each providing a different degree of automation and addressing a different part of the crystallo­graphic workflow (Vonrhein *et al.*, 2007 ▸; Brunzelle *et al.*, 2003 ▸; Minor *et al.*, 2006 ▸; Holton & Alber, 2004 ▸; Kroemer *et al.*, 2004 ▸; Ness *et al.*, 2004 ▸; Terwilliger, 2000 ▸, 2004 ▸).

Table 2 ▸ summarizes these systems and their relation to *Auto-Rickshaw*. The distinguishing emphasis of *Auto-Rickshaw* was web-accessible, decision-driven routing across multiple programs and phasing strategies, with feedback fast enough to support beamline decisions.

## 7. *Auto-Rickshaw* at the beamline

At a synchrotron beamline, time is limited, data quality varies and decisions often need to be made while the experiment is still in progress. The beamline version of *Auto-Rickshaw* connected processed data to automated phasing and preliminary model building, helping users decide whether to continue collecting data, continue measurements at another wavelength of the radiation, collect a derivative, increase multiplicity or move to another sample.

Two representative beamline examples illustrate this point. In the first case, reported as real case R7 in the original *Auto-Rickshaw* paper (Panjikar *et al.*, 2005 ▸), a selenium-labelled protein diffracting to 2.55 Å resolution with high solvent content was solved sufficiently rapidly to influence the ongoing experiment. This case corresponds to the aryl-alcohol oxidase structure from *Pleurotus eryngii*, later deposited as PDB entry 3fim (Fernández *et al.*, 2009 ▸). After substructure solution, the *Auto-Rickshaw* decision makers selected *SHELXE* for phase calculation and continued with automated model building in *ARP*/*wARP*. The Beamline Version produced an interpretable map and a partial α-helical model with 363 of 593 residues built while data collection was still in progress; at that point, the user halted collection of the second wavelength of a planned MAD experiment because the space-group ambiguity had already been resolved and the solvent content correctly estimated. The Advanced Version subsequently built 87% of the model. The difference between the 593 residues described for the R7 case and the 566 residues reported in the deposited structure reflects poorly ordered regions in the final deposited coordinate model. This example shows how *Auto-Rickshaw* could demonstrate that fewer data than originally planned were sufficient for structure solution and the valuable beamtime saved could be used for other experiments.

A complementary beamline scenario illustrates the opposite outcome. In a second heavy-atom case, initial SAD, two-wavelength MAD and three-wavelength MAD attempts did not produce a solution. A successful structure-solution route was obtained only after higher redundancy anomalous data had been collected. Although this example was reported as an operational beamline/teaching case rather than as a separate structure report, it captures an important use of automated feedback: *Auto-Rickshaw* could indicate that the current data were insufficient and that additional data, rather than continuation of the original strategy, were required (Panjikar, 2010 ▸).

These representative scenarios are summarized in Table 3 ▸. This feedback addressed practical questions faced during an experiment and also recorded the programs used, the phasing route selected and key intermediate decisions. At the end of a run, *Auto-Rickshaw* listed the relevant publications to cite for the software and protocols used, helping users prepare the structure-solution description for publication.

As synchrotron MX moved towards automated sample handling, remote access and high-throughput data collection, this role became more valuable. Structural genomics programmes, fragment-screening projects and mail-in crystallography all created pressure for reproducible, scalable and minimally supervised workflows. *Auto-Rickshaw* lowered the barrier to experimental phasing, but its output still required crystallographic judgement: models, maps, substructures and failed runs all had to be interpreted.

## 8. Scientific use and community impact

The scientific impact of *Auto-Rickshaw* is reflected not only in its methodological role, but also in its sustained use by the macromolecular crystallography community. By providing a web-accessible and decision-driven structure-solution platform, *Auto-Rickshaw* lowered the barrier to experimental phasing and enabled users to test multiple phasing routes without having to install, configure and manually coordinate a large number of crystallographic programs.

Cumulative server and citation statistics for the period from 2005 to August 2023 provide a useful measure of this community use and are summarized in Table 4 ▸. The data demonstrate that *Auto-Rickshaw* became a practical resource for both routine and challenging structure-solution problems across a large international user base. The distribution of submitted jobs illustrates the practical breadth of the platform: SAD and MR accounted for the largest fractions of use, while MRSAD, MAD, S-SAD and RIP protocols provided important additional routes for more specialized or difficult cases.

These statistics should therefore be interpreted as evidence of broad community adoption rather than as a direct count of unique biological projects. The publication record reflects sustained impact, with the platform and its major methodological extensions generating significant citations. Together, the usage and citation records show that *Auto-Rickshaw* has functioned both as a software service and as a crystallographic methodology that influenced automated experimental phasing, MR-assisted phasing and beamline-oriented structure solution.

### 8.1. Broader scientific and biopharmaceutical impact

The contribution of *Auto-Rickshaw* extended beyond methods development and individual proof-of-principle applications. During the structural genomics era, automated phasing was important for converting the large numbers of diffraction datasets generated by high-throughput programmes into interpretable electron-density maps and initial models. The MRSAD protocol, for example, was developed and evaluated using ten Joint Center for Structural Genomics cases, demonstrating how weak anomalous information could complement incomplete molecular-replacement models (Panjikar *et al.*, 2009 ▸). Its use was not confined to these test cases. A search of the Protein Data Bank for depositions that explicitly record *Auto-Rickshaw* as structure-solution or phasing software identifies at least 282 entries at the time of writing. These include structures produced by the Joint Center for Structural Genomics, Midwest Center for Structural Genomics, Northeast Structural Genomics Consortium, Structural Genomics Consortium and Center for Structural Genomics of Infectious Diseases. Because software provenance has not always been recorded comprehensively in PDB depositions, this number is likely to represent a lower bound.

The deposited structures and their associated publications demonstrate a wide biological range. Applications in infectious-disease research include the Ebola virus interferon antagonist VP24, Marburg virus VP40, the Zika virus NS5 methyltransferase, a *Legionella* superbinding SH2 domain and the ferric preacinetobactin receptor BauA from the opportunistic pathogen *Acinetobacter baumannii* (Zhang *et al.*, 2012 ▸; Oda *et al.*, 2016 ▸; Coloma *et al.*, 2016 ▸; Kaneko *et al.*, 2018 ▸; Moynié *et al.*, 2018 ▸). Other examples include proteins involved in bacterial signalling and biofilm formation, DNA and RNA recognition, restriction-endonuclease activity, chromosome organization and membrane transport (Schlundt *et al.*, 2017 ▸; Tamulaitiene *et al.*, 2014 ▸, 2017 ▸, 2019 ▸; Schneider *et al.*, 2019 ▸; Chen *et al.*, 2021 ▸). The corresponding PDB entries show that *Auto-Rickshaw* was applied to targets ranging from small soluble enzymes to multidomain, membrane-associated and protein–nucleic acid systems.

Several applications are also directly relevant to therapeutic discovery. Structures determined with the assistance of *Auto-Rickshaw* have supported the analysis of parasite-specific proteins and drug targets, including *Plasmodium* profilin, plasmepsins IX and X, *Helicobacter pylori* hypoxanthine–guanine–xanthine phosphoribosyltransferase and prolyl-tRNA synthetase complexes investigated as potential inhibitors of toxoplasmosis (Kursula *et al.*, 2008 ▸; Hodder *et al.*, 2022 ▸; Keough *et al.*, 2021 ▸; Yogavel *et al.*, 2023 ▸). The wider enzyme portfolio includes proteins involved in amino-acid and cofactor biosynthesis, plant metabolism and natural-product biosynthesis. These examples illustrate how automated structure solution can provide the structural starting point for mechanistic interpretation, target validation and structure-guided ligand development.

The scientific impact of *Auto-Rickshaw* was therefore not limited to the individual algorithms assembled within the pipeline. By providing experimental and hybrid phasing through a web-accessible workflow, checking alternative solution strategies automatically and returning interpretable maps and models without requiring users to master each underlying program, it lowered practical and computational barriers for crystallographers who were not specialists in phasing. This principle continues in work adapting automated structure-determination approaches to protein–ligand analysis and fragment-based drug-discovery pipelines (Khandokar *et al.*, 2023 ▸).

## 9. Evolution: MRSAD, RIP, S-SAD and multi-dataset workflows

The history of *Auto-Rickshaw* after its initial release is a history of adaptation to crystallographic practice. As experimental methods evolved and user needs changed, the platform incorporated additional protocols and refinements that extended it beyond the initial SAD, MAD, SIRAS and MR workflows.

The development of MRSAD was one of the most significant extensions of *Auto-Rickshaw* (Panjikar *et al.*, 2009 ▸). The combined MR/MRSAD workflow is illustrated in Fig. 3 ▸. The conceptual basis for combining molecular-replacement phases with weak anomalous information had been demonstrated earlier by Schuermann and Tanner, who showed that sulfur anomalous differences collected at Cu *K*α wavelength could help overcome model bias in MR structure determination (Schuermann & Tanner, 2003 ▸). The *Auto-Rickshaw* implementation extended this idea into an automated decision-driven workflow. It responded to the increasing availability of homologous structures and to the practical reality that many molecular-replacement solutions are correct but only partially useful. A search model may provide enough phase information to guide density modification, but not enough to produce an interpretable map or reliable automated model building. Conversely, the anomalous signal may be too weak to solve the structure independently. By combining imperfect model-derived phases with anomalous differences, MRSAD formalized a hybrid strategy in which the two sources of phase information reinforce one another within an automated workflow.

A demanding application of this strategy was the B–C protein complex from the *Yersinia entomophaga* ABC toxin system, originally reported as a large 2.5 Å resolution crystal structure in *Nature* (Busby *et al.*, 2013 ▸). A subsequent methodological analysis described how cross-crystal averaging and *Auto-Rickshaw* MRSAD were combined to solve the 4354-amino-acid structure under challenging phasing conditions (Busby *et al.*, 2016 ▸).

Radiation-induced phasing (RIP), molecular-replacement-assisted RIP (MRRIP) and UV-induced RIP represented another form of methodological expansion. The conceptual basis for RIP was established by Ravelli and coworkers, who showed that specific radiation damage could be used as a source of phase information for solving macromolecular crystal structures (Ravelli *et al.*, 2003 ▸). This concept was subsequently extended to ultraviolet-induced structural changes, where UV exposure could generate site-specific differences suitable for phasing (Nanao & Ravelli, 2006 ▸). Later work showed that ultraviolet radiation could also generate useful, site-specific intensity changes in selenomethionine-containing protein crystals while retaining sufficient isomorphism for phasing. In particular, UV-induced cleavage around selenium sites enabled UV-RIP phasing of SeMet proteins (Panjikar *et al.*, 2011 ▸), and related UV-RIPAS experiments combined UV-induced isomorphous differences with anomalous signal to produce phase sets comparable to those from conventional MAD approaches (de Sanctis *et al.*, 2011 ▸). These protocols exploited radiation damage not only as a limitation of data collection but as a possible phasing signal. This inversion of perspective, turning an experimental complication into useful crystallographic information, fits naturally with the *Auto-Rickshaw* philosophy of exploring multiple possible routes to structure solution.

A subsequent structure determination of snakin-1 provides a useful example of RIP phasing in practice (Yeung *et al.*, 2016 ▸). In this case, molecular replacement, *ab initio* attempts and an *Auto-Rickshaw* MAD strategy were unsuccessful, but radiation-damage differences between datasets enabled the automated RIP protocol of *Auto-Rickshaw* to solve the structure. *Auto-Rickshaw* then produced a largely traced model after *SHELXE* phasing, *ARP*/*wARP* model building and further density modification, as described in the supporting information to Yeung *et al.* (2016 ▸).

Sulfur-SAD also became increasingly important as synchrotron beamlines, detectors and data-collection strategies improved. Native sulfur phasing is attractive because it avoids derivatization or selenomethionine labelling, but it requires accurate measurement of weak anomalous differences. An important early demonstration was provided by Dauter and coworkers, who showed that the anomalous signal from S atoms could be used as a practical tool for protein structure solution, including lysozyme data collected at Cu *K*α wavelength near 1.54 Å (Dauter *et al.*, 1999 ▸). Automation can help by consistently evaluating the signal strength, testing appropriate resolution limits and applying robust downstream phasing procedures.

Multi-dataset workflows were another important area of development. Fast pixel-array detectors, brighter microfocus beamlines and automated sample changers made it practical to collect many partial or complete datasets from one or more crystals. Useful signal may be distributed across multiple crystals, multiple sweeps or multiple related datasets, so automated systems must manage not only a single set of reflections, but also the selection, comparison and combination of datasets. This motivated multiple-dataset modes in *Auto-Rickshaw*, including approaches in which datasets were selected, clustered or combined according to unit-cell similarity, isomorphism, radiation damage and anomalous signal before being passed into SAD, SIRAS or MAD phasing workflows (Panjikar *et al.*, 2015 ▸, 2017 ▸). These developments extended the original single-job structure-solution concept towards beamline-scale data evaluation and automated selection of useful datasets.

## 10. Current status and outlook of the *Auto-Rickshaw* web service

The original EMBL Hamburg *Auto-Rickshaw* web service remained available for many years and supported a large number of external users, jobs and structure-solution projects. After the relocation of the principal developer from EMBL Hamburg to the Australian Synchrotron in June 2011, the public web service continued to be maintained remotely while further beamline-focused development proceeded at the Australian Synchrotron.

In August 2023, the public EMBL Hamburg web service was discontinued. Sustaining such a structural biology-oriented computational web service required a specialized combination of software-development, systems-administration, crystallographic and beamline expertise. By 2023, this complete support structure was no longer available locally at EMBL Hamburg. The discontinuation should therefore be understood as a change in service hosting and support capacity, rather than as a loss of scientific value or technical relevance of the pipeline.

At present, *Auto-Rickshaw* is available for macromolecular crystallography users at the Australian Synchrotron MX beamlines. By 2021, it had been installed on the ASCI cluster using Docker and Kubernetes for high-throughput job launching, enabling scalable execution from MX beamline computers through command-line operation, a web-based graphical interface and automatic data-processing workflows (Panjikar, 2021 ▸). This deployment also extended the use of the platform towards multiple-dataset evaluation, ligand-binding analysis and fragment-screening workflows.

A future goal is to identify a sustainable hosting model through which *Auto-Rickshaw* can be made available in its fullest capacity to a wider structural biology community. Such a model would need to balance open scientific access with modern requirements for cybersecurity, software maintenance, user management, licencing of external crystallo­graphic programs and reproducible deployment. The recent *ASWEBRICK* and Docker-based developments are practical steps towards that goal.

## 11. From *Auto-Rickshaw* to *ASWEBRICK*: secure and reproducible deployment

A recent stage in the evolution of *Auto-Rickshaw* is the transition from a classical web-based structure-solution service to secured, containerized and beamline-integrated deployment. Automated structure-solution software increasingly forms part of an integrated beamline, data-management and decision-support environment rather than only a post-experiment analysis tool.

The evolution of *Auto-Rickshaw* deployment modes is summarized in Table 5 ▸ and illustrated in Fig. 4 ▸. The *ASWEBRICK* development represents a further step in this direction. Rather than treating *Auto-Rickshaw* only as a single public web service, *ASWEBRICK* emphasizes secure server operation, controlled user access and compatibility with institutional computing and cybersecurity requirements. In parallel, Docker-based deployment provides a portable environment in which the *Auto-Rickshaw* pipeline and *pyAR* (the Python scripting layer of *Auto-Rickshaw*) can run reproducibly while established crystallographic programs are accessed through controlled external software paths. This separation between the pipeline environment, user data and crystallographic software improves maintainability and makes the workflow more suitable for local, cluster-based and teaching deployments.

The inclusion of *AlphaFold*-derived or related search models is a natural extension, allowing modern model-prediction methods to enter a decision framework originally developed for experimental phasing and molecular replacement.

## 12. *Auto-Rickshaw* in the current landscape

The current structural biology landscape differs substantially from that of 2005. Detectors are faster, beamlines are more automated, remote and mail-in access are routine, fragment-screening and serial approaches generate large numbers of datasets, and computational infrastructure increasingly includes clusters, containers and cloud services. Molecular replacement has also become more powerful, aided by the growth of structural databases and, more recently, high-quality predicted models such as those generated by *AlphaFold* (Jumper *et al.*, 2021 ▸).

Predicted structural models can now serve as search models within the molecular-replacement branch of the pipeline. When a suitable model is available, it can provide initial phases that may be combined with experimental information or passed through density-modification, model-building and refinement steps. Because this implementation has not yet been formally published, it is best regarded here as an emerging extension rather than as a fully documented protocol.

Recent developments in *SHELXE* reinforce this connection. Modern *SHELXE* workflows explicitly consider phases derived from partial or predicted models, and include approaches to reduce model bias during tracing and density modification (Usón & Sheldrick, 2024 ▸). This provides a conceptual link between *AlphaFold*-assisted molecular replacement, model-bias control and the earlier *Auto-Rickshaw* strategy of combining imperfect model-derived phases with experimental phasing information.

A further modern application area is fragment and ligand screening, where large numbers of protein–ligand datasets must be analysed rapidly. Work at the Australian Synchrotron has explored how automatic structure-determination approaches derived from *Auto-Rickshaw* can be adapted to streamline fragment-based drug-discovery pipelines and protein–ligand complex analysis (Khandokar *et al.*, 2023 ▸).

These changes do not remove the need for experimental phasing pipelines. Difficult cases still involve weak anomalous signals, multiple datasets, radiation damage, partial models, low-resolution data and ambiguous maps. Modern pipelines may use containers, cloud execution, predicted models and machine-learning classifiers, but they still need to convert imperfect information into interpretable structural models.

The rise of AI-assisted crystallography gives this question renewed importance. Machine learning is already influencing several stages of the workflow, including crystal centring, diffraction-image classification and data reduction, model prediction, electron-density map interpretation and decision support (Ito *et al.*, 2019 ▸; Ke *et al.*, 2018 ▸; Jumper *et al.*, 2021 ▸; Dialpuri *et al.*, 2024 ▸; Vollmar & Evans, 2021 ▸). In this context, the evolution of *Auto-Rickshaw* illustrates how explicit domain knowledge, established algorithms and transparent diagnostics can be organized into a reliable decision-making workflow.

Looking ahead, an *Auto-Rickshaw*-style platform could combine rapid multi-dataset assessment with predicted search models, experimental anomalous evidence and automated model-bias checks, selecting among these sources according to their measured reliability rather than treating any one source as definitive (Jumper *et al.*, 2021 ▸; Usón & Sheldrick, 2024 ▸). Machine-learning components could assist with classification and prioritization, while explicit crystallographic criteria and auditable intermediate results preserve scientific transparency. Secure containerized deployment could make the same validated workflow available at beamlines, institutional clusters and teaching facilities, and could support high-throughput applications such as fragment and ligand screening (Panjikar, 2021 ▸; Khandokar *et al.*, 2023 ▸). The central future challenge is therefore not simply to add more algorithms, but to integrate new sources of structural information without losing reproducibility, diagnostics or expert oversight.

## 13. Lessons from *Auto-Rickshaw*’s design philosophy

Several lessons emerge from the history of *Auto-Rickshaw*. Useful automation must be crystallographically informed: real diffraction data require conditional logic rather than a fixed list of commands. Robust defaults also matter, because many users need a scientifically reasonable first answer without losing expert control.

The platform also shows that software interoperability is a scientific capability. *Auto-Rickshaw* connected specialized programs through file conversion, input generation, output parsing, error handling, job control and result presentation. Finally, automation is most valuable when it remains transparent. Users need to know which route was taken, which programs were used, what intermediate statistics were obtained and why the calculation succeeded or failed.

## 14. Conclusions

*Auto-Rickshaw* was developed to guide experimental data through the decision landscape of macromolecular structure solution. Its historical importance lies not only in the number of phasing protocols it supported, but in the way it joined beamline needs, crystallographic algorithms, software engineering and expert decision making.

The integration of *SHELXC*, *SHELXD* and *SHELXE* gave *Auto-Rickshaw* a powerful experimental phasing core, while the broader pipeline connected this core to molecular replacement, density modification, automated model building and refinement. Extensions such as MRSAD, RIP, MRRIP, S-SAD and multiple-dataset workflows show how the platform evolved with the needs of the crystallographic community.

A short history of *Auto-Rickshaw* is therefore also a history of a larger transition: from manual execution of specialized programs towards adaptive, reproducible and beamline-aware structure-solution workflows. As structural biology moves towards unattended data collection, cloud computation, predicted models, electron-microscopy map interpretation and AI-assisted decision making, the principles embodied by *Auto-Rickshaw* remain relevant.

## Figures and Tables

**Figure 1:**
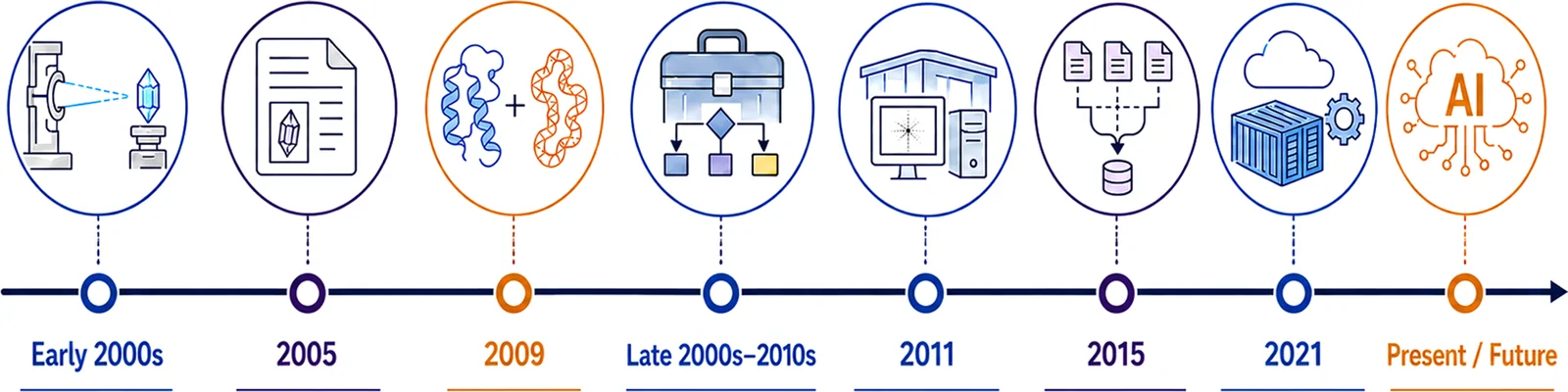
Historical timeline of *Auto-Rickshaw*. The timeline illustrates key milestones in the evolution of *Auto-Rickshaw* from its EMBL Hamburg/DESY beamline origins in the early 2000s through modern secure and containerized deployment. Major developments include the 2005 initial release, the introduction of MRSAD in 2009, relocation to the Australian Synchrotron in 2011 and containerized deployment by 2021. A detailed description of each phase is provided in the text.

**Figure 2:**
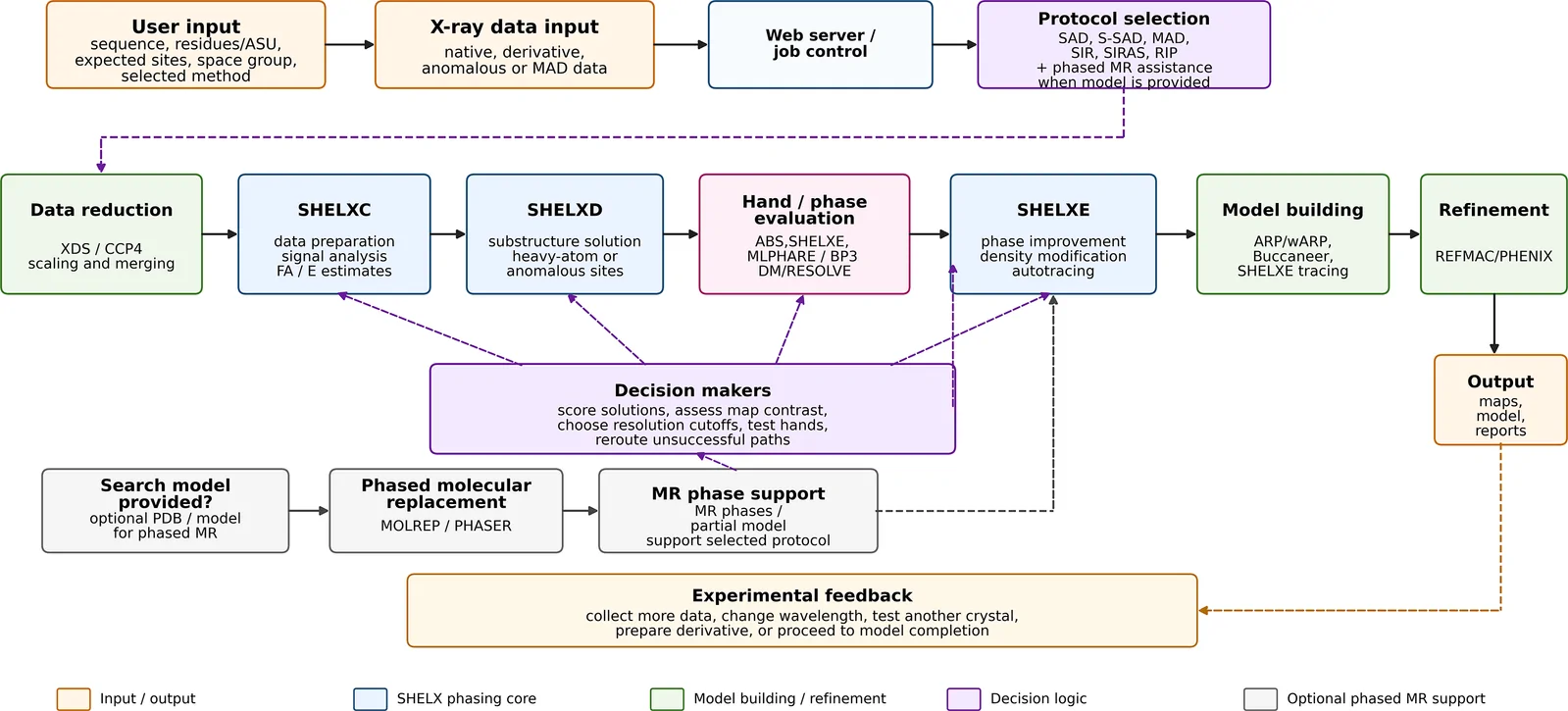
Experimental phasing workflow in *Auto-Rickshaw*. User input and diffraction data are passed through the web-server/job-control layer to protocol selection, which determines the appropriate data-reduction and phasing route. The central *SHELX*-based path uses *SHELXC* for data preparation and signal analysis, *SHELXD* for anomalous or heavy-atom substructure solution and *SHELXE* for hand evaluation, phase improvement, density modification and autotracing. Decision makers monitor intermediate results, test alternative resolution cutoffs and hands, and reroute unsuccessful paths. When a search model is provided, phased molecular replacement is carried out using *MOLREP* or *Phaser*. The resulting MR phases or partial model can assist the selected experimental phasing protocol before phase improvement, density modification and autotracing in *SHELXE*. Final maps, models and diagnostic reports inform subsequent experimental decisions.

**Figure 3:**
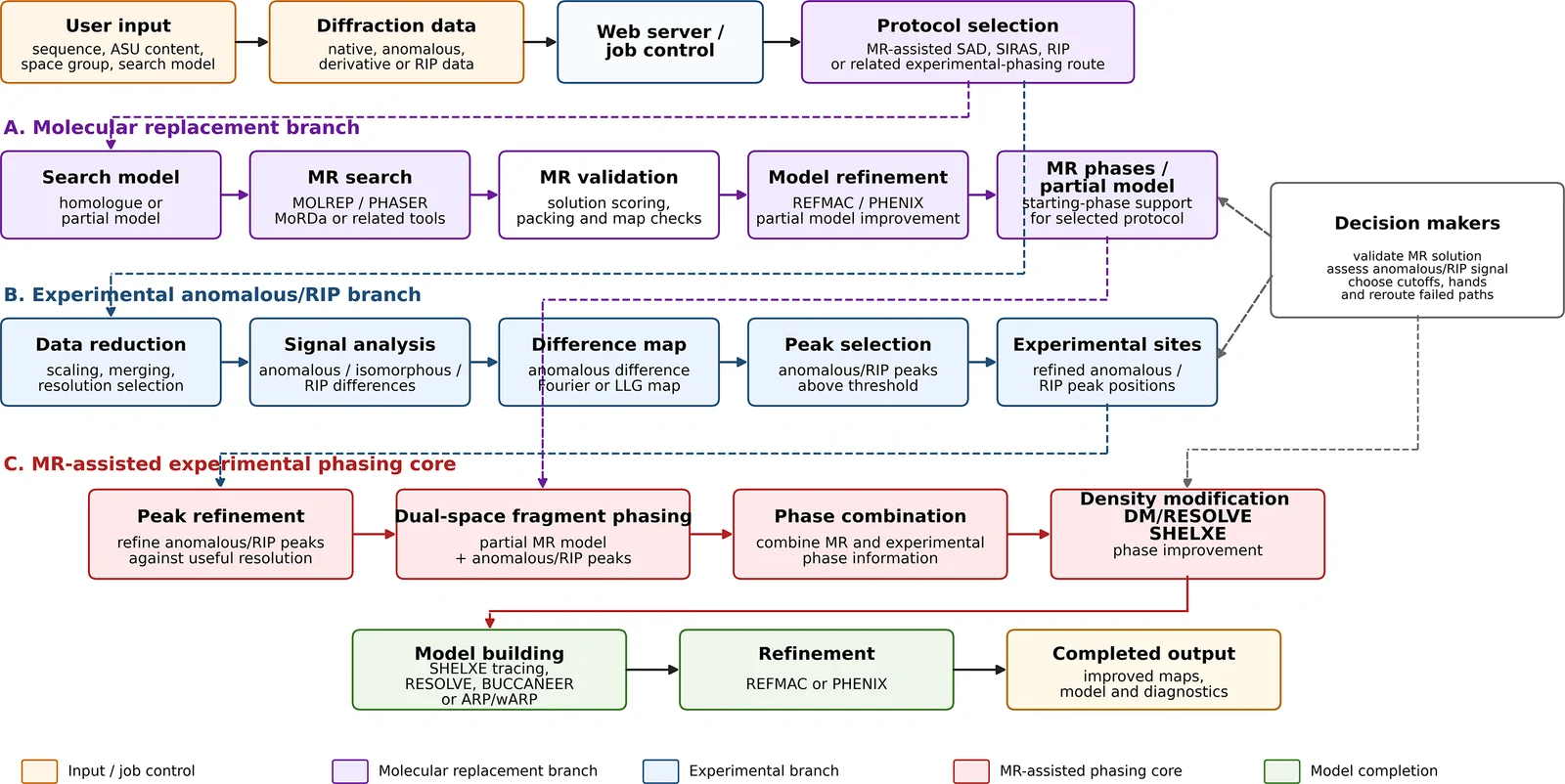
MR/MRSAD phasing protocol in *Auto-Rickshaw*. The workflow combines a molecular-replacement branch, in which a search model is used to obtain and validate an MR solution, with an experimental anomalous, isomorphous or RIP branch, in which signal analysis and difference-map interpretation identify useful experimental sites. The MR-derived phases or partial model and the experimental sites are combined in the MR-assisted experimental phasing core, followed by dual-space fragment phasing, phase combination, density modification, model building and refinement.

**Figure 4:**
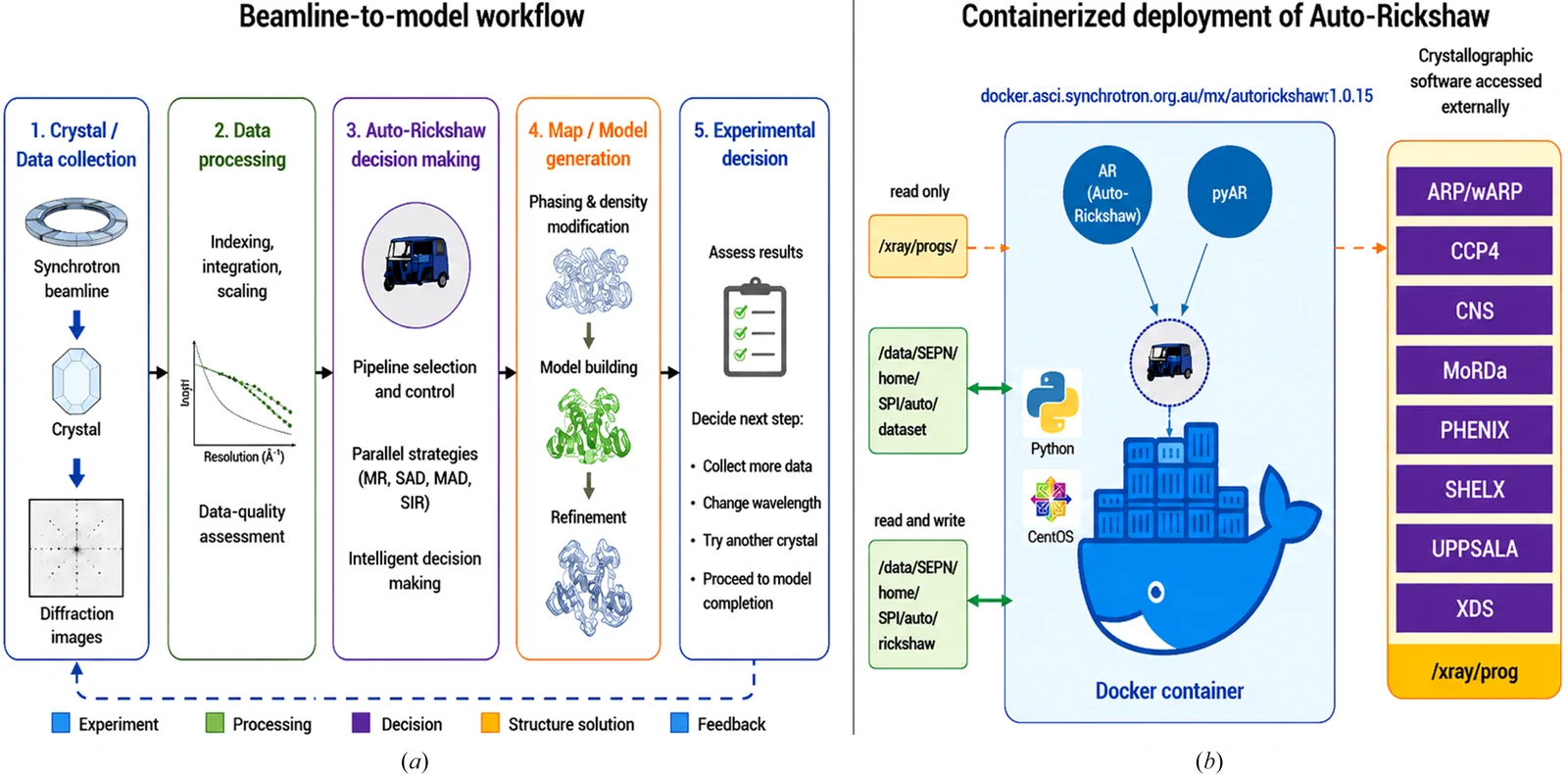
*Auto-Rickshaw* as a bridge between beamline experiment and structure solution. (*a*) Conceptual beamline-to-model workflow. Diffraction data collected at a synchrotron beamline are processed and assessed before entering the *Auto-Rickshaw* decision-making layer, where appropriate phasing, density-modification, model-building and refinement strategies are selected and controlled. The resulting maps, models and diagnostic reports feed back into experimental decisions, including whether to collect additional data, change wavelength, test another crystal or proceed to model completion. (*b*) Modern containerized implementation of *Auto-Rickshaw*. Beamline or user data directories are mounted into the Docker container, separating read-only input data from read–write output directories. The *Auto-Rickshaw *and *pyAR* layers operate within a controlled software environment, while established crystallographic programs are accessed externally through a read-only software tree. This arrangement improves reproducibility, maintainability and deployment at synchrotron facilities.

**Table 1:** Representative phasing strategies supported by *Auto-Rickshaw* and the principal role of *SHELX* programs in the workflow Abbreviations are defined in the main text.

Method	Key *SHELX* programs	Role in *Auto-Rickshaw*
SAD	*SHELXC*, *SHELXD*, *SHELXE*	Primary experimental phasing from anomalous signal
S-SAD	*SHELXC*, *SHELXD*, *SHELXE*	Native phasing from weak sulfur anomalous signal
MAD	*SHELXC*, *SHELXD*, *SHELXE*	Multi-dataset anomalous phasing near an absorption edge
SIR/SIRAS	*SHELXC*, *SHELXD*, *SHELXE*	Heavy-atom derivative phasing using isomorphous and/or anomalous differences
MR	*SHELXE*	Model-based initial phasing followed by phase improvement and model building
MRSAD	*SHELXC*, *SHELXD*	Hybrid use of partial MR phases and anomalous differences
RIP/MRRIP	*SHELXC*, *SHELXD*, *SHELXE*	Phasing from radiation-induced differences, optionally assisted by MR

**Table 2:** Selected automated structure-solution systems contemporary with *Auto-Rickshaw*

System	Main emphasis	Relation to *Auto-Rickshaw*
*autoSHARP*	Automated heavy-atom refinement and experimental phasing around the *SHARP* framework	Related experimental phasing automation; more focused around the *SHARP* phasing environment
*ACrS*	Automated crystallographic system for high-throughput protein structure determination	Service-oriented automation of structure-determination steps
*HKL*-3000	Integrated data reduction, scaling, structure solution and initial model building, linking diffraction-image processing with downstream crystallographic interpretation (Minor *et al.*, 2006 ▸)	Provides a highly integrated route from diffraction images to an initial model; *Auto-Rickshaw* instead emphasizes web-accessible, decision-driven routing after processed reflection data have entered the phasing pipeline
*ELVES*	Automated structure determination including data-processing and downstream structure-solution steps	A broad multi-step automation effort, including the data-processing side
*APRV*	Automated data processing, refinement and visualization	Focused on automated processing/refinement/visualization workflows
*CRANK*	Automated experimental phasing within the *CCP*4 framework	Shared experimental phasing automation goals, especially for SAD/MAD/SIRAS workflows
*SOLVE*/*RESOLVE*	Automated heavy-atom solution, phasing, density modification and model building	Key early demonstration of automated experimental structure solution and model building
*Auto-Rickshaw*	Web-accessible, multi-program, decision-driven pipeline for experimental phasing, MR-assisted phasing, model building, refinement and beamline feedback	Distinctive emphasis on beamline validation, adaptive decision making and routing across multiple external crystallographic programs

**Table 3:** Representative beamline decision-support scenarios enabled by *Auto-Rickshaw*

Beamline scenario	*Auto-Rickshaw* feedback	Experimental consequence
Fewer data sufficient	A selenium-labelled protein dataset collected at the peak wavelength was sufficient for rapid phasing and initial model building	Further planned data collection at the inflection wavelength could be halted, saving beamtime and allowing the user to proceed to further experiments
More data needed	Initial SAD, two-wavelength MAD and three-wavelength MAD attempts did not yield a solution for a heavy-atom derivative case	Additional high-redundancy anomalous data were required, leading to a successful phasing strategy
Methods-style output	The pipeline recorded the selected route, programs used, intermediate diagnostics and model-building outcome	Users received not only maps and models, but also a structured record useful for interpretation and publication methods descriptions including the relevant references for the underlying programs used

**Table 4:** Cumulative scientific use and community impact of *Auto-Rickshaw* based on server statistics for the period 2005–August 2023 and citation counts available during manuscript preparation The difference between successful structure-solution runs and nonredundant outcomes reflects the practical use of the server: individual structure projects often involve repeated submissions, alternative protocols or different datasets, so the nonredundant count provides a conservative estimate of unique structure-solution outcomes.

Metric	Cumulative value
Computational tasks submitted	102018
User base	3089
Institutional reach	825
Successful structure-solution runs	19324
Nonredundant structure-solution outcomes	6532
Peer-reviewed citations	758 citations associated with the main *Auto-Rickshaw* publications and major methodological extensions as of June 2026
Foundational paper	Panjikar *et al.* (2005 ▸): 553 citations
MRSAD paper	Panjikar *et al.* (2009 ▸): 205 citations

**Table 5:** Evolution of *Auto-Rickshaw* deployment modes

Deployment mode	Primary use	Historical significance
Original web server	Remote automated structure solution	Made automated phasing, density modification, model building and refinement accessible to the academic crystallographic community
Beamline-integrated version	Rapid feedback during MX data collection	Connected structure-solution attempts directly to experimental decision making at the beamline
*ASWEBRICK* secured server	Controlled institutional deployment	Represents the transition from a general web service to a secured, managed and beamline-compatible crystallographic automation platform
Docker/containerized deployment	Local, cluster, teaching and reproducible use	Improves portability and reproducibility by separating the *Auto-Rickshaw* execution environment from user data and externally maintained crystallo­graphic software
Predicted-model-assisted workflows	Use of *AlphaFold*-derived or related predicted models as search models	Represents an emerging, currently unpublished extension in which modern predicted structures provide starting phases within the existing *Auto-Rickshaw* decision framework
